# Hyperpolarizing DNA Nucleobases via NMR Signal Amplification by Reversible Exchange

**DOI:** 10.3390/molecules28031198

**Published:** 2023-01-25

**Authors:** Bryce E. Kidd, Max E. Gemeinhardt, Jamil A. Mashni, Jonathan L. Gesiorski, Liana B. Bales, Miranda N. Limbach, Roman V. Shchepin, Kirill V. Kovtunov, Igor V. Koptyug, Eduard Y. Chekmenev, Boyd M. Goodson

**Affiliations:** 1 School of Chemical & Biomolecular Sciences, Southern Illinois University, Carbondale, IL 62901, USA; 2 Department of Chemistry, University of Tennessee, Knoxville, TN 37996, USA; 3 Department of Chemistry, Biology, and Health Sciences, South Dakota School of Mines & Technology, Rapid City, SD 57701, USA; 4 International Tomography Center SB RAS, 3A Institutskaya St., Novosibirsk 630090, Russia; 5 Department of Chemistry, Integrative Biosciences (Ibio), Karmanos Cancer Institute (KCI) Wayne State University, Detroit, MI 48202, USA; 6 Materials Technology Center, Southern Illinois University, Carbondale, IL 62901, USA

**Keywords:** hyperpolarization, SABRE, PHIP, nucleic acids, NMR & MRI

## Abstract

The present work investigates the potential for enhancing the NMR signals of DNA nucleobases by parahydrogen-based hyperpolarization. Signal amplification by reversible exchange (SABRE) and SABRE in Shield Enables Alignment Transfer to Heteronuclei (SABRE-SHEATH) of selected DNA nucleobases is demonstrated with the enhancement (*ε*) of ^1^H, ^15^N, and/or ^13^C spins in 3-methyladenine, cytosine, and 6-O-guanine. Solutions of the standard SABRE homogenous catalyst Ir(1,5-cyclooctadeine)(1,3-bis(2,4,6-trimethylphenyl)imidazolium)Cl (“IrIMes”) and a given nucleobase in deuterated ethanol/water solutions yielded low ^1^H *ε* values (≤10), likely reflecting weak catalyst binding. However, we achieved natural-abundance enhancement of ^15^N signals for 3-methyladenine of ~3300 and ~1900 for the imidazole ring nitrogen atoms. ^1^H and ^15^N 3-methyladenine studies revealed that methylation of adenine affords preferential binding of the imidazole ring over the pyrimidine ring. Interestingly, signal enhancements (*ε*~240) of both ^15^N atoms for doubly labelled cytosine reveal the preferential binding of specific tautomer(s), thus giving insight into the matching of polarization-transfer and tautomerization time scales. ^13^C enhancements of up to nearly 50-fold were also obtained for this cytosine isotopomer. These efforts may enable the future investigation of processes underlying cellular function and/or dysfunction, including how DNA nucleobase tautomerization influences mismatching in base-pairing.

## 1. Introduction

With an energy difference between nuclear spin states that is much less than the thermal energy, *kT*, NMR accesses only ~10^−3^% of available molecules (e.g., ~0.0032% for ^1^H spins at B_0_ = 9.4 T), and thus suffers from poor detection sensitivity. Hyperpolarization methods [1] such as dynamic nuclear polarization (DNP) [2,3], spin-exchange optical pumping (SEOP) [4,5], and parahydrogen-induced polarization (PHIP) [2,6,7] produce highly non-Boltzmann spin populations to greatly enhance NMR signals. With hyperpolarization, the rapid NMR signal acquisition of less-sensitive and low-natural-abundance nuclei (e.g., ^15^N, ^13^C, ^129^Xe, etc.) is greatly facilitated, making it feasible to perform the NMR/MRI of low-concentration species. Among these hyperpolarization techniques, PHIP-based approaches [6,7,8] are attractive because they can be conducted rapidly with low operational costs, merely requiring access to parahydrogen gas (*p*-H_2_, a nuclear spin isomer of ordinary molecular hydrogen), a catalyst, and an appropriate external magnetic field.

**Figure 1:**
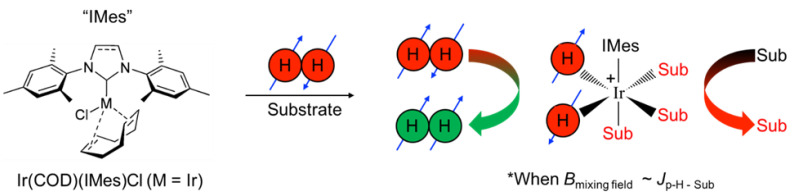
In the presence of *p*-H_2_ and substrate, a pre-catalyst [e.g., Ir(1,5-cyclooctadeine)(1,3-bis(2,4,6-trimethylphenyl)imidazolium)Cl, Ir(COD)(IMes)Cl)] first undergoes a 4- to 6-coordinate transformation induced by the initial exposure to *p*-H_2_, giving rise to the standard “IrIMes” SABRE catalyst [17]. The formation of a transient hexa-coordinate center, wherein the symmetry of the nascent hydride spins is broken, allows spin order to be transferred to reversibly ligating substrates through the J-coupling network, particularly within a mixing field (B_mixing field_) that roughly matches the frequency difference between source (parahydrogen-derived hydrides) and target spins (protons or other spin−1/2 nuclei of the exchangeable substrate) to the magnitude of the J coupling.

One such PHIP-based approach is signal amplification by reversible exchange (SABRE) [9,10]. Unlike hydrogenative PHIP [8], which requires the pairwise addition of *p*-H_2_ atoms across an unsaturated chemical bond, SABRE does not result in permanent chemical change of the substrate. Instead, SABRE involves the transient binding of the substrate and *p*-H_2_ to the catalyst to enable the transfer of spin order to the substrate’s nuclear spins through the J-coupling network, allowing bulk hyperpolarization of the “free” substrate to build up over time with subsequent exchange (Figure 1). Unlike ^1^H SABRE, hyperpolarization of nuclei with lower gyromagnetic ratios (e.g., non-quadrupolar ones like ^15^N and ^13^C) usually does not suffer from strong inter/intramolecular dipolar interactions, and thus often decays over longer time scales [11,12,13,14]; long-lived spin states can offer even longer hyperpolarization lifetimes [15]. Whereas the field-matching condition for ^1^H SABRE is in the mT regime, the efficient SABRE of heteronuclei requires microtesla fields [11,16], and thus is commonly performed within a magnetic shield with a constant or variable internal field, i.e., SABRE-SHEATH (SABRE in SHield Enables Alignment Transfer to Heteronuclei) [16].

SABRE can thus produce solution-phase hyperpolarization for various applications, including the creation of biologically-friendly hyperpolarized spectral probes for NMR, and metabolic contrast agents for MRI [13,14,18,19,20,21,22,23,24]. However, despite its advantages, a persistent challenge of the methodology has been the limited range of applicable substrates due to the necessity of requiring an *sp-* and *sp*^2-^hybridized lone pair to interact with the catalyst’s Ir-metal center, initially limiting SABRE mostly to nitrogen heterocycles [2,9,11,12,16]. While a number of approaches have recently been developed to greatly widen SABRE’s scope [18,19,20] (including biologically relevant carboxylic acid derivatives like α-ketoglutarate and pyruvate) [21,22,25,26,27,28], the myriad biologically relevant NHCs nevertheless make this structural motif of continued interest for SABRE targets for both fundamental studies and envisioned applications [23].

**Figure 2:**
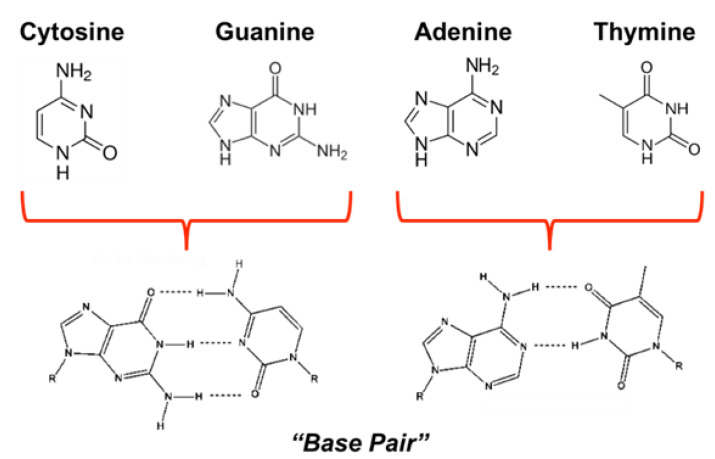
Structures of primary DNA nucleobases that form base pairs through hydrogen-bonding.

Of the NHCs studied thus far, derivatives of purine comprise the most widely occurring NHC family in nature and consist of fused pyrimidine and imidazole rings [29,30,31]. Moreover, derivatives of purine and pyrimidine, such as the nucleobases adenine, thymine, guanine, and cytosine, comprise DNA and participate in specific hydrogen bonding to complete canonical (A:T/G:C) Watson-Crick base pairing [32] (Figure 2). The resulting primary nucleic acid sequence gives rise to secondary structures composed of supercoils that result from protein complexes wound about a DNA helix [33,34]. These structures, along with other modifications (e.g., methylation, histone modification, etc.), are responsible for epigenetic gene regulation [34,35], which has far-reaching consequences in both normal (e.g., cell differentiation) and pathogenic development (e.g., cancer) [34,36,37]. This type of control primarily dictates which segments of the genetic sequence are available for reading (thereby acting as on-and-off switches [38]), depending on the shape, spacing, and composition of the helix and associated further levels of the structure. With RNA these effects are further compounded by the additional functions of RNA, ranging from signaling to catalysis [39,40].

Interestingly, nucleobase tautomers play a key role in epigenetic control, as they can alter the secondary level of the DNA’s structure [41,42]. For example, tautomerization of a nucleobase can lead to distortions in the shape of (or spacing between) DNA strands, which are then targeted by DNA repair enzymes. In some cases, an enzyme may fail to initiate the repair, thus epigenetically leading to silencing or changes in the gene’s expression [42], with implications for cancer and other diseases—depending on the location of the tautomerization [35]. Thus, any method that can probe epigenetic interactions has potential value in both clinical and research settings [35,37].

Due to the ubiquity of the nucleobases that comprise DNA and RNA, along with the myriad of other functions that nucleobases perform (e.g., cell signaling [43,44,45] and ATP [46]), such compounds are ripe for investigation by methods that may be able to sensitively and non-invasively report on nucleobase interactions in living systems or biological media. Such developments would be particularly relevant if those methods could complement the capabilities of other bioanalytical techniques under development (e.g., Refs. [43,47]). Given that the detection of nucleobase-containing biomarkers of disease have opened new areas of research, there should be natural interest in the investigation of rapid and inexpensive parahydrogen-based methods for hyperpolarizing nucleobases and their use in potential applications; however, to date there have only been a few such studies reported. In one type of approach, the unique resonances of catalyst-bound hyperpolarized hydrides [48,49,50] are used to detect the presence of specific nucleobases (and their tautomers) with high sensitivity, including in complex biological media [51,52]. Hövener et al. reported ^1^H hyperpolarization of adenine and adenosine as part of efforts to demonstrate continuous polarization in MRI applications [53]. More recently, as part of a larger effort to demonstrate SABRE enhancement in a large number of different molecules, Colell et al. [12] showed ^15^N enhancement of ^15^N-labelled adenine (ε~200) as an example where enamine-imine tautomers are sensitive to SABRE-SHEATH [12]; this sensitivity may be exploitable in potential biological applications. Finally, work has also been performed to optimize ^1^H and ^15^N hyperpolarization of pyrimidine, which is the framework for cytosine and thymine [54]. In the present work, we explore the applicability of SABRE and SABRE-SHEATH to various nucleobases, motivated by the desire to expand the current scope of these approaches to support the future development of new techniques for studying various biological systems and diseases—both for cellular studies and ultimate potential in vivo applications.

## 2. Materials and Methods

**Figure 3:**
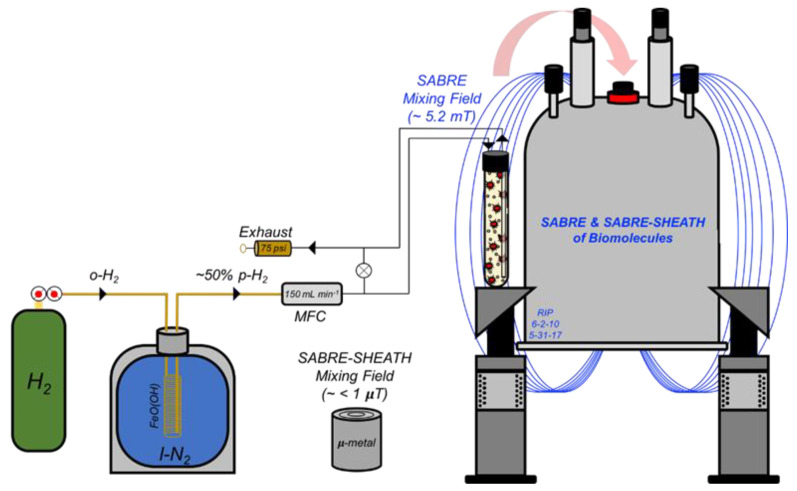
^1^H SABRE and ^15^N/^13^C SABRE-SHEATH setup (used for experiments at SIUC). Hydrogen gas (H_2_) from a cylinder (with initial *p*-H_2_ fraction of 25%) is passed over a catalyst bed of FeO(OH) cooled in liquid nitrogen for ~50% conversion to *p*-H_2_. A mass flow controller (MFC) at 150 mL min^−1^ allows for the precise regulation of *p*-H_2_ bubbling rate within the NMR tube; pressure was typically maintained at 75 psi. ^1^H SABRE and ^15^N/^13^C SABRE-SHEATH experiments were performed by bubbling *p*-H_2_ through the sample while placed in either the NMR magnet’s fringe field (~5 mT) or a mu-metal magnetic shield (~1 µT), respectively, prior to rapid manual transfer of the sample to the 9.4 T magnet for high-field detection.

The homogenous (Ir(COD)(IMes)Cl, MW = 639.67 g·mol^−1^) pre-catalyst was synthesized as previously described [17]. Each NMR solution consisted of 4 mM catalyst and 40 mM substrate in 600 µL solution of either 100% C_2_D_5_OD (3-methyladenine) or 92% C_2_D_5_OD: 8% D_2_O (cytosine and 6-O-methylguanine); D_2_O was necessary to increase the solubility of the latter nucleobases. Experimental setups at SIUC and Vanderbilt were described previously [11,16,19,55]. Each sample solution was transferred to a 5 mm O.D. NMR tube affixed with a 0.25-inch O.D. Teflon tube, sealed with a wye-connector, and activated at elevated temperatures (60–70 °C) for at least 10 min prior to acquisition. The experimental setup is schematically shown in Figure 3.

Parahydrogen generators used in this work provided either: *p*-H_2_ enrichment of ~50% (at 75 psi and 150 mL min^−1^ bubbling rate or at 75 psi and 110 mL min^−1^ bubbling rate at SIUC and Vanderbilt, respectively); or, ~90% *p*-H_2_ (for some heteronuclear studies at Vanderbilt). Most ^15^N/^13^C SABRE-SHEATH experiments were performed at Vanderbilt University using a µ-metal shield that was degaussed manually with a Variac and degaussing coil prior to use [56]; all of those experiments used medium-wall NMR tubes. All NMR experiments were performed on either an Agilent 400 MHz DD2 spectrometer with a wide-bore actively shielded (Oxford) magnet (SIUC) or a Bruker AVANCE III 400 MHz spectrometer with a narrow-bore actively shielded magnet (Vanderbilt). Single-scan acquisitions with 10°–pulses were used to acquire both SABRE-enhanced ^1^H spectra and ^1^H spectra from thermally polarized samples. Pulses of 90° of 18 µs (^15^N) or 10 µs (^13^C) and 1 scan were used to acquire ^15^N and ^13^C SABRE-SHEATH spectra. ^15^N thermally polarized reference spectra were acquired with a standard 8.65 M ^15^N_2_-imidazole solution (in D_2_O) using a 90°–pulse of 18 µs and 250 s pre-acquisition delay time, and one scan. ^13^C thermally polarized reference spectra were acquired with the activated cytosine solution at 70 °C using a 90°–pulse of 18 µs, 250 s delay time, and one scan.

## 3. Results and Discussion

### 3.1. ^1^H SABRE of DNA Nucleobases and Ethanol

**Figure 4:**
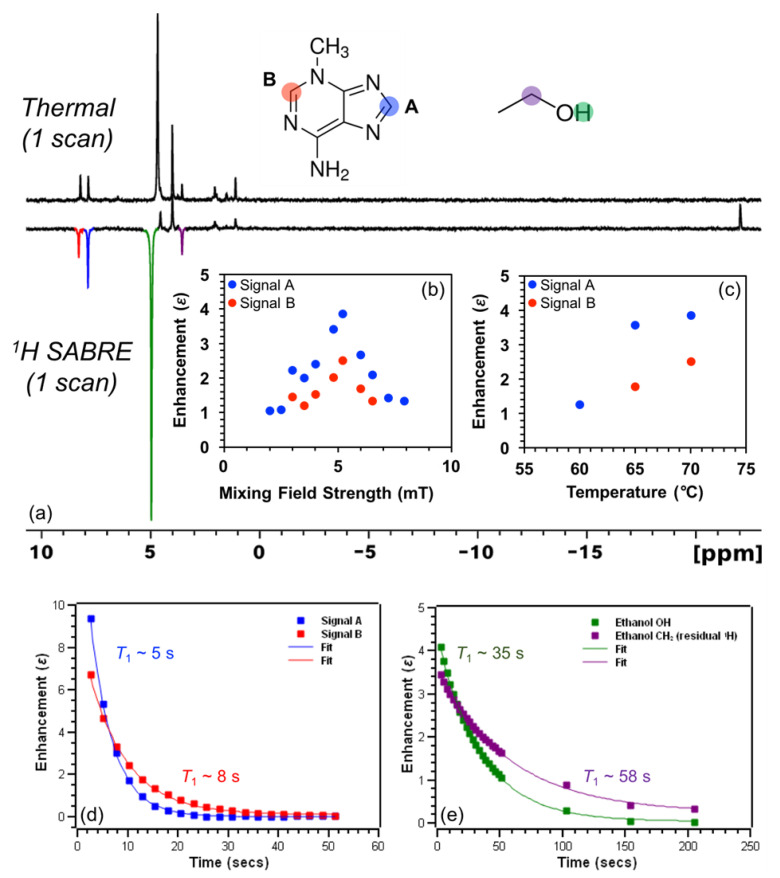
(**a**) ^1^H NMR thermal (*top*) and SABRE enhanced (*bottom*) spectra of 3-methyl adenine at 70 °C show differential enhancement (and presumptive preferential binding) of the imidazole ring with a maximum *ε* of ~(-)4 at (**b**) ~5.2 mT and (**c**) 70 °C. HP ^1^H signals of 3-methyladenine exhibit *T*_1_ time constants (**d**) of ~5 s (^1^H_A_) and ~8 s (^1^H_B_), whereas HP signals from residual ^1^H spins of C_2_D_5_OD solvent molecules decay with *T*_1_ time constants (**e**) of ~35 s (-OH) and ~58 s (-CHD, residual ^1^H). Note that the -OH resonance of ethanol shifts due to a reduction of temperature when the solution is bubbled with *p*-H_2_. *T*_1_ values: in (**d**): 4.52 ± 0.03 (blue); 7.60 ± 0.13 (red); in (**e**): 35.1 ± 0.4 (green); 58.2 ± 1.2 (purple); similar *T*_1_ values were measured at the lower temperatures (not shown).

Here we probe the efficacy of ^1^H SABRE enhancement for the DNA nucleobases 3-methyladenine, 6-O-methylguanine, and cytosine (others were attempted but lacked sufficient solubility under our conditions). We begin by showing ^1^H NMR spectra from thermally polarized and SABRE enhanced 3-methyladenine in 100% C_2_D_5_OD at 70 °C (Figure 4a). Interestingly, both aromatic hydrogens (labelled ^1^H_A_ and ^1^H_B_) are hyperpolarized (albeit weakly), but with ^1^H_A_ exhibiting slightly larger (factor of ~1.5) enhancement over ^1^H_B_, suggestive of preferential binding of the imidazole ring over the pyrimidine ring to the catalyst. Naively, such differential enhancement might have also been explained by differences in relaxation. However, ^1^H_A_ exhibits a *shorter T*_1_ time constant of ~5 s in comparison to ^1^H_B_ (~8 s, albeit measured at high field instead of the mixing field; Figure 4d; at milli-tesla and micro-tesla fields, the relaxation rates may be significantly different [57], although here we expect the qualitative trend to be the same). The faster rate of hyperpolarization decay for ^1^H_A_ presumably reflects greater intermolecular interactions with the catalyst itself (which can act as a relaxation agent) [58], as well as possible contributions from hydrogen-bonding interactions with C_2_D_5_OD in the complex (discussed below).

The present 3-methyladenine enhancement pattern is the reverse of that observed by Hövener et al. in adenine, which included a larger enhancement for the pyrimidine ^1^H_B_ resonance (by a factor of approximately two) compared to that of the imidazole ^1^H_A_ spin [53]. Such behavior might be rationalized by the fact that 3-methyladenine has one fewer available *sp*^2^-hybridized nitrogen atoms on the pyrimidine ring to bind to the catalyst; on the other hand, Hövener et al. also observed a larger enhancement for the imidazole resonance in adenosine despite the increased sterics likely caused by the sugar moiety [53]. More generally, binding with the 5-membered ring might be expected to be preferred because of the reduced sterics/stronger binding of 5-membered rings (e.g., imidazole) compared to 6-membered rings (e.g., pyridine). For example, high ^15^N polarization values in excess of 50% were reported for methylated imidazole with -CH_3_ in the *ortho*- position in the case of metronidazole [13,14,59], but no detectable ^15^N polarization (*P*_15N_ < 0.01%) was seen for methylated pyridine in the ortho- position in the case of 2-picoline [60]. ^1^H SABRE of 3-methyladenine as a function of mixing field strength (Figure 4b) shows a maximum enhancement for both signals at ~5.2 mT, which was then selected as the mixing field for the remaining ^1^H SABRE experiments presented here. ^1^H SABRE of 3-methyladenine as a function of temperature (60–70 °C, Figure 4c) shows an increase in enhancement with temperature, which can be attributed to the better matching of exchange rates of 3-methlyadenine and *p*-H_2_ with the catalyst, once the temperature was raised sufficiently to help mitigate the otherwise poor solubility of the substrate in 100% C_2_D_5_OD below 60 °C.

**Figure 5:**
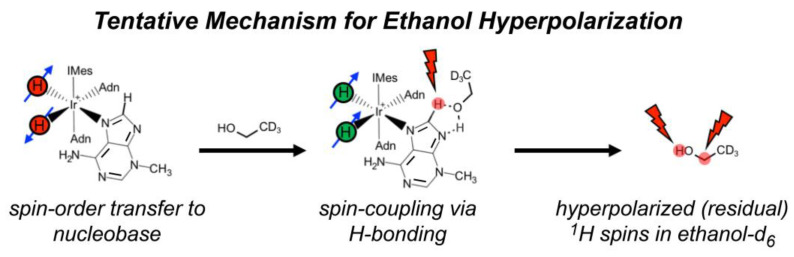
Illustration of the proposed mechanism for hyperpolarization of residual protons of deuterated ethanol solvent molecules. In this scenario, bound 3-methyladenine becomes hyperpolarized, deuterated ethanol (with residual ^1^H spins) hydrogen-bonds to 3-methyladenine, and then the solvent molecule becomes hyperpolarized via spin-relayed SABRE.

Figure 4a also reveals a weak ^1^H SABRE of the resonance from residual -OH (slightly shifted due to temperature drop during *p*-H_2_ bubbling) and -CHD (residual ^1^H) of the bulk solvent, C_2_D_5_OD. There have been only a few previous studies of hyperpolarized alcohol solvents via SABRE (e.g., Ref. [61]). Currently, there are three proposed mechanisms for hyperpolarization of solvent alcohol molecules: (1) hyperpolarization through direct coordination to the metal center; (2) A SABRE-RELAY [18] type mechanism (particularly in a slightly acidic environment), wherein a hydrogen ion transfers to free substrate (post hyperpolarization), is hyperpolarized from spin coupling to the substrate protons, and then exchanges with the alcohol group on the solvent; and (3) a solvent molecule hydrogen-bonds to a free nitrogen of a (e.g., catalyst-bound) substrate, allowing the spin order to transfer via the extended scalar coupling network. Given that ^1^H_A_ shows greater enhancement over ^1^H_B_, the imidazole ring of 3-methyladenine contains two nitrogen atoms, and only a single Ir-hydride signal is present (−22 ppm), we suggest that the ethanol solvent molecules may become hyperpolarized predominantly through hydrogen-bonding to 3-methyladenine while bound at the metal-center (Figure 5); direct binding may also contribute, though it is expected that SABRE-RELAY is unlikely to be a significant contributor because of the relatively low ^1^H enhancements observed for the substrate.

The shorter *T*_1_ time constant of ^1^H_A_ for 3-methyladenine (Figure 4d) may reflect intermolecular interactions, which may include exchange with hydrogen-bonding solvent molecules. Interestingly, deuterated ethanol residual -OH and -CHD spins exhibit hyperpolarization decay (*T*_1_) time constants of ~35 s and ~58 s at 70 °C (Figure 4e), respectively, which is substantially longer than the ^1^H’s of 3-methyladenine. For residual ^1^H of C_2_D_5_OD, the contribution from intra- and intermolecular dipole-dipole interactions to *T*_1_ relaxation are minimized since the gyromagnetic ratio of ^2^H is ~6.5 times smaller than ^1^H. The shorter *T*_1_ for the residual -OH resonance likely reflects the greater participation in exchange.

**Figure 6:**
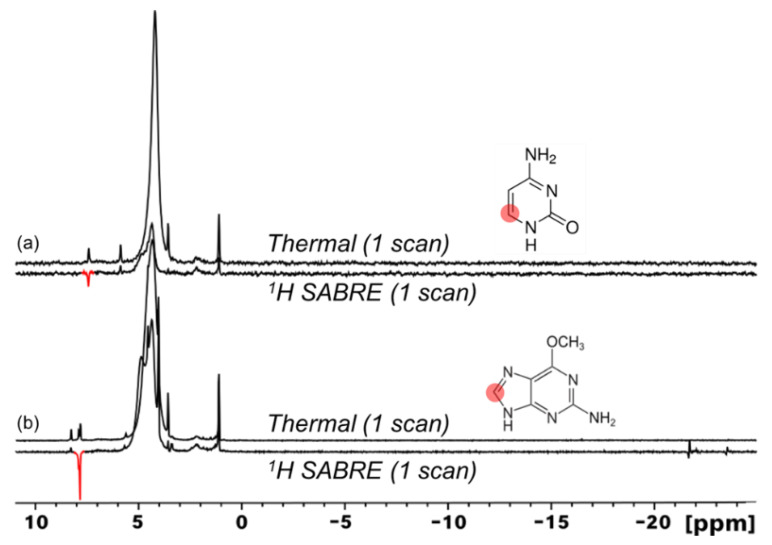
^1^H SABRE spectra of cytosine ((**a**), *amino* tautomer shown) and 6-O-methylguanine (**b**) show minimal hyperpolarization of ^1^H nuclei adjacent to binding nitrogen atoms. In (**b**), the bottom spectrum is scaled by a factor of ~1.9 compared to the top spectrum.

Expanding the scope of ^1^H SABRE to other DNA nucleobases, we also studied cytosine (Figure 6a) and 6-O-methylguanine (Figure 6b). Small but clear ^1^H SABRE enhancements were observed for ^1^H resonances of both of these substrates. The weakness of the effects can partly be attributed to the need for 8% D_2_O to help solubilize the substrates, even at elevated temperatures; the use of aqueous media is challenging for SABRE due to the poor solubility of H_2_ in water and a reduced substrate exchange rate with the catalyst [62,63,64,65,66,67,68], thus often resulting in lower SABRE enhancements.

Analogous to 3-methyladenine, 6-O-methylguanine appears to show preferential catalyst binding of the imidazole ring, likely due to less steric hindrance. Hydride resonances showed generally weaker signals compared to those obtained with 3-methyladenine (Figure 4a), particularly with cytosine, where virtually no detectable hydride resonances were observed. The weaker hydride signals are generally consistent with lower SABRE enhancements. Moreover, whereas 3-methyladenine gave rise to a single hydride peak (indicating magnetic equivalence of the complex’s two hydride sites on the NMR time scale), the multiple (weak) hydride signals observed with 6-O-methylguanine (comprising an asymmetric, antiphase doublet and a weak absorptive singlet) likely indicate the simultaneous presence of at least two different hydrogenated metal complexes (i.e., having different ligands) in significant concentration (see, for example, Refs. [20,22]). Note also the lack of SABRE enhancement of residual solvent resonances. Tautomerization has been suggested to result in a decrease in ^1^H enhancements [69]. Indeed, tautomerization, along with elevated temperatures [70,71] and the weak binding of these substrates, likely contributes to the weakness of the ^1^H SABRE effects observed for this set of molecules. Nonetheless, we have shown successful ^1^H SABRE for three nucleobases, including the DNA base cytosine and the modified base 6-O-methylguanine for the first time.

### 3.2. ^15^N and ^13^C SABRE-SHEATH of Nucleobases

**Figure 7:**
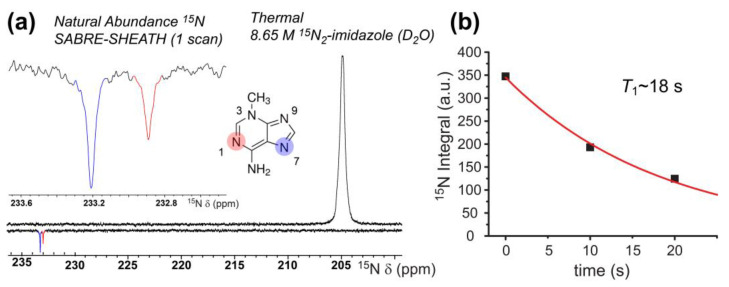
(**a**) Natural-abundance ^15^N SABRE-SHEATH of 40 mM 3-methyladenine (*bottom*) reveals *ε* values of ~3300 (^15^N_A_) and ~1900 (^15^N_B_), respectively, when compared to a thermally polarized signal from a ^15^N_2_-imidazole reference sample (*top*); a close-up of the enhanced resonances is shown in the *inset*. Tentative ^15^N shift assignments were made in light of Refs. [72,73,74]. (**b**) Hyperpolarization decay curve obtained from single-point acquisitions with varying delay at 9.4 T prior to acquisition (the fit was constrained by including a fourth point set to zero at 1000 s).

While ^1^H SABRE represents a rapid screening method for molecules of interest (and was used as such here), ^1^H hyperpolarization can suffer rapid *T*_1_ decay due to strong inter- and intramolecular interactions. Non-quadrupolar heteronuclei (e.g., ^15^N and ^13^C) suffer weaker dipolar interactions and thus generally allow greater accumulation and retention of hyperpolarization because of longer *T*_1_ values. Moreover, accessing such nuclei allows for nuclei-specific investigations into biological processes. ^15^N and ^13^C SABRE-SHEATH of DNA nucleobases may thus prove to be more useful in evaluating tautomerization dynamics—in addition to potentially enabling future applications. Here we begin by showing natural-abundance ^15^N (0.365%) SABRE-SHEATH of 3-methyladenine at 70 °C in 100% C_2_D_5_OD (Figure 7a). SABRE-SHEATH shows the enhancement of two adjacent ^15^N NMR signals from two nitrogen atoms (sites/resonances “A” and “B”) that integrate to a ~2:1 ratio for ^15^N_A_ compared to ^15^N_B_, which when compared to the thermally polarized signal from ^15^N_2_-imidazole, corresponds to enhancement values of *ε* ~3300 (corresponding to ^15^N polarization just above 1%) and *ε* ~1900, respectively. The ~230 ppm chemical shifts of these two resonances are consistent with the values expected for the N1, N3, and N7 positions of adenine, with the N9 and -NH_2_ positions expected ~160 ppm and ~78 ppm, respectively [72,73,74]. However, no enhancements are observed for the NMR signals in those lower ranges. Moreover, given that no enhancement would be expected for the N3 site in 3-methyladenine (because of steric inhibition of catalyst binding), the enhanced sites are likely associated with the N1 (pyrimidine ring) and N7 (imidazole ring) positions. Moreover, to be consistent with the ^1^H SABRE results in Figure 4a (where the larger ^1^H enhancement was observed on the imidazole proton), we tentatively assign the more-downfield peak (N_A_, with the greater SABRE enhancement) in Figure 7a to N7, and the more-upfield peak (N_B_) to N1. The absence of any signal attributable to the N9 site would be consistent with the prevalence of a tautomer wherein the N9 position is protonated (as it is commonly depicted).

This interpretation of the ^15^N results would be consistent with the ^1^H SABRE results for 3-methyladenine and C_2_D_5_OD (Figure 4), as well as the notion of preferential binding of the imidazole ring to the catalyst. Figure 7b shows a ^15^N *T*_1_ decay curve, wherein a somewhat rapid decay in signal (*T*_1_ = 18.4 ± 1.8 s) is observed. These results represent the first natural abundance ^15^N enhancement (spin concentration: ~150 µM) of a nucleobase via SABRE-SHEATH.

**Figure 8:**
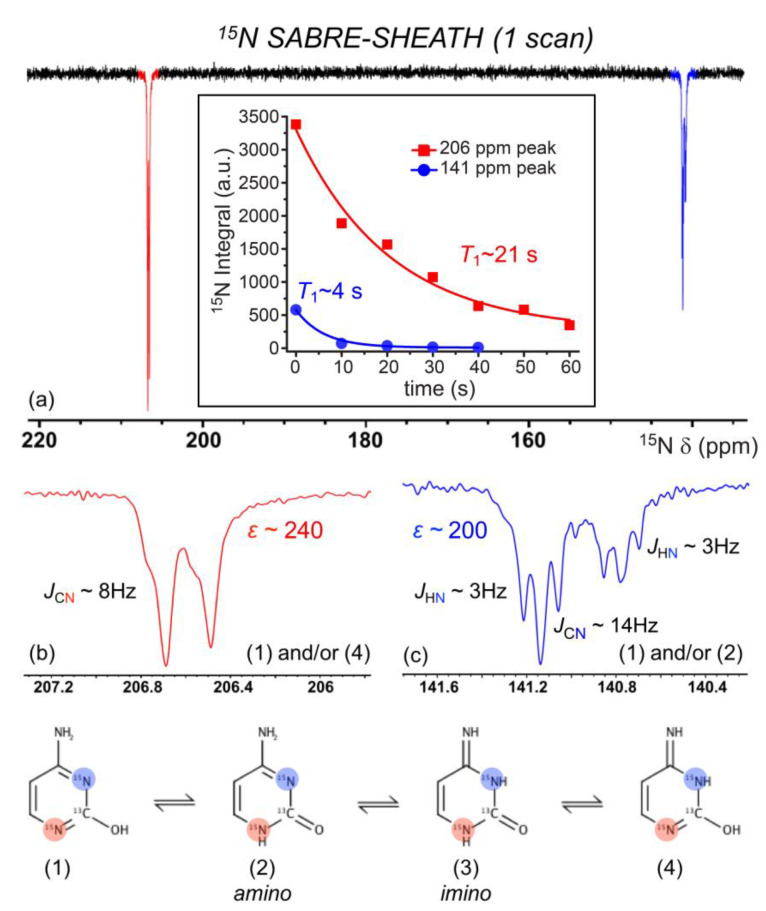
(**a**). ^15^N NMR SABRE-SHEATH of doubly-labeled cytosine appears to reveal the preferential binding of tautomers (1), (2), and/or (4) via ^15^N_A_ (**b**) over ^15^N_B_ (**c**). Steric hindrance from the adjacent ketone and amine to ^15^N_B_ likely reduces the binding ability of tautomers (1) and (2). Enhancements calculated from the 8.65 M ^15^N_2_-imidazole thermally polarized reference section shown in Figure 7. ^15^N spectra here were taken without ^1^H decoupling. The *inset* of (**a**) shows that the N_A_ (206 ppm) and N_B_ (141 ppm) sites decay with different ^15^N *T*_1_ values (measured at 9.4 T; each point is a separate experiment with variable delay time at high field prior to acquisition) of 4.3 ± 0.5 s (blue) and 20.6 ± 4.2 s (red).

We also investigated the ^15^N SABRE-SHEATH of doubly-labelled cytosine (2-^13^C; 1,3-^15^N_2_). The enhancement of cytosine ^15^N signals from a 40 mM solution (92% C_2_D_5_OD: 8% D_2_O) at 70 °C is shown in Figure 8a, following polarization transfer in a mixing field of ~1 µT for 10 s; a short bubbling time was used to prevent a rapid decrease in temperature that could cause the substrate to crash out of the solution. In the figure, we see a modest selectivity in relative enhancement (1:0.75) for ^15^N_A_ (206 ppm) to ^15^N_B_ (141 ppm) compared to expectations based on a thermally polarized spectrum from a sample containing only labelled cytosine (i.e., without catalyst) with relative integrals of 1:1 (not shown); the absolute enhancements were ~240-fold and ~200-fold, respectively. A *T*_1_ measurement (performed as part of a different experimental run; discussed further below) saw an even-greater variance in the intensity of the two sites (Figure 8a *inset*).

Such deviation may suggest the preferential binding of specific tautomers of cytosine (Figure 8). For both ^15^N atoms to become hyperpolarized, tautomerization of the labile ^1^H about the ketone must take place to generate both *sp*^2^-hybridized ^15^N atoms. Thus, tautomers (1), (2), and/or (4) (Figure 8) can potentially bind to the catalyst. We suggest that the larger *ε* of ^15^N_A_ over ^15^N_B_ is due to preferential binding of (1) and/or (4) to ^15^N_A_ because of steric hindrance from the adjacent ketone and amine to ^15^N_B_, thus reducing interactions of ^15^N_B_ with the catalyst. This is further supported by our weak but non-zero ^1^H SABRE for the adjacent ^1^H (Figure 6). The signal at ~206 ppm (N_A_) shows what appears to be two overlapping doublets that are not well-resolved, each with a *J*-coupling of ~8 Hz that likely arises from *J*_CN_ for that ^15^N coupled to the labeled ^13^C site. Interestingly, the signal at ~141 ppm (N_B_) shows two unique triplets (split by a presumed *J*_CN_ for that site of ~14.5 Hz). Previous work by Shchepin et al. on the sensitivity of ^15^N of imidazole to pH revealed a ~30 ppm up-field shift in the ^15^N resonance as pH is varied from 1 to 12, thus reporting on protonated and unprotonated ^15^N [75]. The finer splitting arises from *J*_NH_; the above *J* assignments are consistent with thermally polarized ^1^H-decoupled ^15^N experiments (not shown), where the decoupling collapsed the ~3 Hz splittings but not the larger (unequal) splittings. The ^15^N relaxation measurements performed at 9.4 T (Figure 8a *inset*) showed that the N_A_ site had a similar high-field *T*_1_ (20.6 ± 4.2 s) as that of the enhanced ^15^N site in 3-methyladenine. However, the N_B_ site’s hyperpolarization decayed much more quickly (*T*_1_ = 4.3 ± 0.5 s), suggesting the contribution of additional relaxation mechanisms for this spin (e.g., greater contributions from exchange) that could serve to exacerbate the selective ^15^N SABRE enhancement.

**Figure 9:**
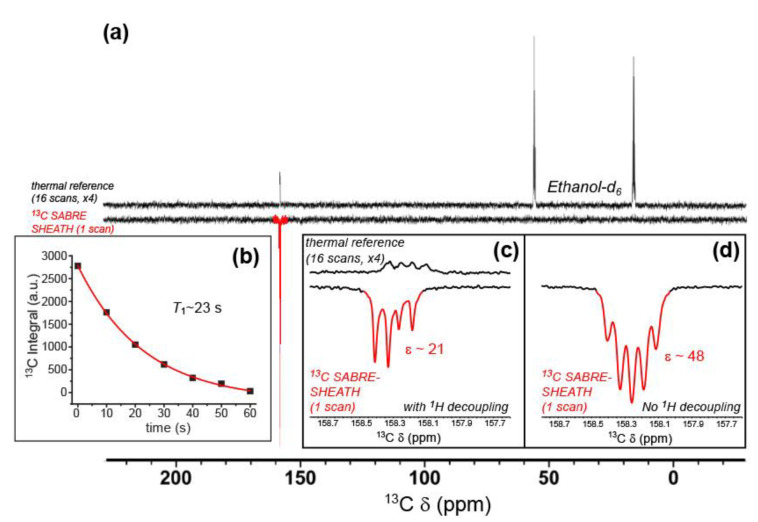
(**a**) ^13^C NMR SABRE-SHEATH of doubly-labeled cytosine (*bottom*), compared to a thermally polarized reference scan from the same sample (16 scans, *top*). Close-ups of the cytosine resonances from these spectra, both taken under conditions of broadband ^1^H-decoupling, are shown in (**c**); enhancement (*ε*) ~21-fold. (**b**) The hyperpolarization decay curve for the integrated cytosine carbonyl ^13^C resonance, obtained from a second set of SABRE-SHEATH experiments (measured at 9.4 T; each point is a separate experiment with variable delay time at high field prior to acquisition), yielding *T*_1_ of 22.9 ± 0.8 s. Data points were taken from spectra obtained without ^1^H decoupling, an example of which is shown in (**d**); *ε*~48-fold; that spectrum also has a very weak, broad peak at 150 ppm (not shown) that is tentatively assigned to cytosine bound to the catalyst.

Finally, we present cytosine ^13^C enhancements via SABRE-SHEATH. Figure 9a shows a ^13^C SABRE-SHEATH spectrum from doubly-labeled cytosine obtained under conditions of ^1^H decoupling, compared to a thermally polarized reference spectrum from the same sample. A close-up of these spectra is shown in Figure 9c. There, the spectra manifest a non-first-order doublet of doublets arising from the two *J*_CN_ couplings to N_A_ and N_B_. Although the four peaks exhibit equal intensities for the ^13^C thermally polarized spectrum, the SABRE-SHEATH spectrum shows a highly asymmetric pattern of enhancement, which likely arises from unequal efficiencies of polarization transfer through the level anti-crossing regime achieved while the sample was in the magnetic shield. Taken together, the average enhancement (integrating over all four peaks) is ~21-fold. In another series of experiments, ^13^C SABRE-SHEATH spectra were obtained from doubly-labeled cytosine, but without ^1^H decoupling (Figure 9b,d). For these experiments, symmetric multiplets were observed (Figure 9d) with average effective splittings of ~6–8 Hz, reflecting both *J*_CN_ and *J*_CH_ contributions. The larger ^13^C enhancements observed (up to nearly ~50-fold) likely reflect the improved experimental efficiency of polarization transfer and are likely unrelated to the decoupling condition. A series of such spectra were obtained with a variable delay at 9.4 T prior to acquisition, allowing the high-field ^13^C hyperpolarization lifetime to be measured for the ^13^C cytosine resonance (*T*_1_ = 22.9 ± 0.8 s).

A summary of the measurements obtained in this work—SABRE enhancements and *T*_1_ relaxation measurements—is contained below in Table 1. Taken together, the hyperpolarization lifetimes of the heteronuclei measured for these nucleobases is shorter than may be expected. However, when contemplating the rapid *T*_1_ decays for hyperpolarized ^15^N and ^13^C signals of the present nucleobases, it should be noted that chemical shift anisotropy (CSA) is likely to be a significant contributor to *T*_1_ relaxation of ^15^N and ^13^C in these systems. Because CSA relaxation is a function of the square of field strength, SABRE-hyperpolarized DNA nucleobases may indeed prove useful at lower field strengths (e.g., clinical MRI or benchtop NMR), wherein greater enhancements and longer *T*_1_ time constants are likely.

It is also worth noting that the order-of-magnitude lower ^15^N SABRE-SHEATH enhancements for (doubly-labeled) cytosine compared to (naturally abundant) 3-methyladenine are qualitatively consistent with the ^1^H SABRE results for these molecules (Figure 4 and Figure 6). Such weaker ^15^N enhancements would also be much more difficult to observe (and hence optimize) with naturally abundant cytosine. Moreover, it may be difficult to achieve large ^13^C SABRE-SHEATH enhancements in nucleobases without the ^15^N labeling of the nitrogen sites, because abundant quadrupolar ^14^N spins can greatly accelerate ^13^C relaxation in the magnetic shield due to scalar relaxation of the second kind [76].

## 4. Conclusions

In this study, we have expanded the accessibility of SABRE and SABRE-SHEATH of DNA nucleobases by demonstrating NMR signal enhancement of: ^1^H spins in 3-methyladenine, cytosine, and 6-O-guanine; ^15^N spins in 3-methyladenine and cytosine; and ^13^C spins in cytosine. ^1^H SABRE generally exhibited weak enhancements for each studied nucleobase. However, ^1^H SABRE studies of 3-methyladenine revealed that simple chemical modification (compared to previous work on adenine) gave rise to preferential binding of the imidazole ring over the pyrimidine ring. Moreover, ^1^H SABRE also showed hyperpolarization of the solvent molecules (residual protons of deuterated ethanol), ostensibly through hydrogen-bonding to 3-methyladenine, with *T*_1_ values approaching ~60 s; the proposed mechanism will be explored in more detail with future studies. Preferential binding of the imidazole ring is further supported by the natural-abundance ^15^N SABRE-SHEATH enhancement of 3-methyladenine, upwards of *ε*~3300—the first natural-abundance result for a DNA nucleobase. Finally, ^15^N and ^13^C enhancements of cytosine (upwards of *ε*~240 and *ε*~50, respectively) via SABRE-SHEATH suggest a balance between tautomerization and catalyst binding kinetics. It is likely that significantly larger enhancements could be achieved by utilizing more-recently optimized hyperpolarizer platforms and conditions [22], greater *p*-H_2_ enrichment (in some cases), and alternative pulse sequences designed to improve SABRE-SHEATH efficiency [77]. Thus, while DNA nucleobases may represent a family of challenging SABRE substrates, these results help pave the way for a variety of envisioned biological studies in the future, including efforts to further the fundamental understanding of the interplay of nucleobase tautomerization, base pairing, and disease. For example, hyperpolarized nucleobases may be useful in cellular or in vivo cell-signaling studies. It may also become possible to probe how certain genetic sequences may be more vulnerable to tautomerization (i.e., via mutagenic exposure) in cellular/cell-lysate studies (e.g., by hyperpolarizing one or more nucleotides within a short DNA sequence and exposing it to a tautomerizing agent). Moreover, mismatches in base-pairing could potentially be investigated by unzipping the strand at elevated temperatures, hyperpolarizing the nucleobases, and allowing the strand to anneal upon rapid cooling. Thus, polarization transfer to pairing nucleobases may allow the study of mutagenesis on a molecular level. Indeed, a clear understanding of interactions between SABRE-sensitive and insensitive nucleobases, followed by their various derivatives including nucleotides, may eventually culminate into the hyperpolarization of single- and double-stranded DNA.

## Figures and Tables

**Table 1:** Summary of nucleobase SABRE enhancements and *T*_1_ values.

Nucleobase Variant	Isotope	Resonance/ Assignment *	Maximum Enhancement |e|	*T*_1_ (s) ^†^
3-methyladenine	^1^H	imidazolic, ~7.9 ppm	~9.4 ^‡^	4.52 ± 0.03
	^1^H	pyrimidinic, ~8.2 ppm	~6.7 ^‡^	7.60 ± 0.13
	^15^N	‘^15^N_A_’, N7 *, imidazolic, 233.2 ppm	~3300	18.4 ± 1.8
	^15^N	‘^15^N_B_’, N1 *, pyrimidinic, 232.8 ppm	~1900	
6-O-Methyl- guanine	^1^H	imidazolic, ~7.9 ppm	<2	n.d.
Cytosine	^1^H	Pyrimidinic, ~7.5 ppm	<1	n.d.
Cytosine, doubly labeled (^15^N, ^13^C)	^15^N	‘^15^N_A_’, 206 ppm	~240	20.6 ± 4.2
	^15^N	‘^15^N_B_’, 141 ppm	~200	4.3 ± 0.5
	^13^C	158.3 ppm	~48 (~21, with ^1^H decoupling on)	22.9 ± 0.8

* Tentative assignments shown; ^†^ All *T*_1_ values were obtained at 9.4 T with catalyst; see text for more conditions; ^‡^Obtained from the *T*_1_ experiments; n.d. = not determined.

## Data Availability

Data is available upon request to the corresponding author.
